# Lysophosphatidic acid: a promising biomarker for diagnosing sepsis and predicting in-hospital mortality

**DOI:** 10.3389/fimmu.2025.1725394

**Published:** 2026-01-06

**Authors:** Xiaojuan Li, Tiewei Li, Pengfei Xuan, Hongyan Wang, Jingping Yang

**Affiliations:** 1 Department of Clinical Laboratory, Zhengzhou Key Laboratory of Children’s Infection and Immunity, Henan Children’s Hospital, Children’s Hospital Affiliated to Zhengzhou University, Zhengzhou, Henan, China; 2 Respiratory and Critical Care Medicine Department, Inner Mongolia Baogang Hospital, Baotou, Inner Mongolia, China

**Keywords:** sepsis, lysophosphatidic acid, biomarker, diagnosis, in-hospital mortality

## Abstract

**Background:**

Lysophosphatidic acid (LPA) has anti-inflammatory and protective effects in sepsis, yet clinical evidence on its correlation with sepsis progression and outcomes is limited. This study aimed to evaluate the association of plasma LPA levels with sepsis development, severity, and mortality.

**Methods:**

A total of 42 sepsis patients and 29 controls with common infections were included. Among the sepsis patients, 15 succumbed during hospitalization. Plasma LPA levels were measured, and clinical data were retrospectively analyzed.

**Results:**

Plasma LPA was significantly lower in sepsis patients compared to controls, and further reduced in non-survivors. Notably, correlation analyses suggested that LPA levels were negatively correlated with neutrophil count, procalcitonin, interleukin-6, and Sequential Organ Failure Assessment (SOFA) score. Multivariate regression analysis identified LPA as an independent risk factor for sepsis onset and in-hospital mortality. Receiver operating characteristic (ROC) curve analysis revealed that LPA had a high diagnostic accuracy for sepsis (area under the ROC curve [AUC] = 0.92, 95% CI = 0.86–0.99, P < 0.001) and was a strong predictor of in-hospital mortality (AUC = 0.86, 95% CI = 0.76–0.97, P < 0.001).

**Conclusion:**

Reduced plasma LPA levels in sepsis patients are inversely correlated with infection/inflammation markers and SOFA scores. Together, these results suggest that LPA may serve as a potential diagnostic and prognostic biomarker for sepsis, supporting its potential as a complementary tool to enhance early risk stratification and guide bedside clinical decision-making.

## Introduction

Sepsis is a critical, life-threatening condition caused by a dysregulated immune response to infection; it largely contributes to high morbidity rates and mortality (1). Annually, millions of individuals develop sepsis, which poses considerable challenges to healthcare systems owing to its complex pathophysiology and associated high treatment costs (2–4). Blood culture remains the gold standard for sepsis diagnosis. Nevertheless, this method is constrained by delayed turnaround times, susceptibility to contamination, and low positive pathogenic detection rates. Collectively, these limitations contribute to diagnostic inaccuracies and clinical mismanagement (5, 6). Timely and accurate diagnosis of sepsis is paramount for initiating evidence-based interventions, facilitating effective therapeutic stewardship, and mitigating inappropriate antibiotic use (7, 8). Delayed diagnosis has been strongly correlated with poor outcomes, emphasizing novel, highly specific biomarkers to enable early detection and guide clinical management.

Lysophosphatidic acid (LPA) is a bioactive phospholipid that mediates diverse physiological and pathological processes, including the regulation of immune and inflammatory responses (9). Dysregulation of LPA levels and receptor signaling contributes to multiple immune-related and inflammatory illnesses (10, 11). Its role in inflammation is complex, as LPA can modulate the release of inflammatory mediators from various cell types (12). In lipopolysaccharide (LPS)-induced sepsis models among animals, exogenous administration of LPA or LPA receptor agonism suppresses the release of proinflammatory factors, including interleukin-6 (IL-6), tumor necrosis factor-alpha (TNF-α), and monocyte chemoattractant protein-1 (MCP-1). Simultaneously, it enhances interleukin-10 (IL-10) expression, ameliorating acute tissue injury and improving survival outcomes (13–15).

Mechanistic studies have established LPA’s protective role in sepsis; however, clinical studies investigating the role of LPA in human sepsis remain underexplored. Specifically, the association between fluctuating plasma LPA levels and inflammatory responses and disease severity in sepsis is unclear. Moreover, the diagnostic/prognostic utility of LPA as a biomarker lacks clinical validation. Therefore, this study aimed to clinically validate the diagnostic and prognostic value of LPA in a well-defined patient cohort, specifically quantifying changes in plasma LPA levels, examining its relationship with markers of inflammation/disease severity, and evaluating its performance for sepsis diagnosis and in-hospital mortality prediction.

## Methods

### Study design and population

This cross-sectional study was conducted from August 2024 to April 2025. During this period, we consecutively screened and enrolled eligible patients. Patients meeting the clinical diagnostic criteria for sepsis were allocated to the sepsis group. Meanwhile, patients hospitalized with community-acquired pneumonia (CAP) who did not meet the sepsis criteria were consecutively enrolled as the control group. Inclusion in the sepsis group required meeting established sepsis diagnostic criteria, whereas the control group participants had confirmed infections but did not meet the sepsis diagnostic thresholds. The primary observational endpoint was in-hospital mortality, defined as death occurring at least 24 hours after admission and before discharge. All surviving patients were successfully treated and discharged. The exclusion criteria applied to both groups were as follows: (1) incomplete clinical or laboratory documentation (n = 2); (2) concurrent malignancies, hematological disorders, or autoimmune diseases (n = 5); and (3) lack of written informed consent (n = 2). After applying these exclusion criteria, the study ultimately included 42 sepsis patients and 29 control patients. Among the 42 sepsis patients, 15 experienced in-hospital mortality. The protocol was approved by the Medical Ethics Committee of Inner Mongolia Baogang Hospital (Approval No: 2022-MER-110) per the tenets of the Declaration of Helsinki. Written informed consent was obtained from all participants before enrollment.

### Detecting plasma LPA levels

Venous blood samples (4 mL) were collected from all participants into EDTA-containing tubes on the first day of hospitalization. After clotting at room temperature for 30 minutes, the samples were centrifuged at 500g for 10 min. The harvested plasma was aliquoted and stored at -80 °C until analysis. Total plasma lysophosphatidic acid (LPA) levels were quantified using a commercially available competitive enzyme-linked immunosorbent assay (LPA Assay Kit II, Echelon Biosciences), following the manufacturer’s instructions. This assay utilizes a monoclonal antibody (504B3/B3) with high specificity for LPA and minimal cross-reactivity with related lipids. The detailed step-by-step ELISA protocol, along with comprehensive information on antibody specificity and cross-reactivity, is provided in the **Supplementary Materials**. LPA concentrations in patient samples were determined by interpolating absorbance values (450 nm) against a standard curve. All biomarker measurements, including LPA, were performed using blood samples collected within 24 hours of hospital admission.

### Data collection

Clinical and laboratory data, including age, sex, body mass index (BMI), body temperature, respiratory rate, heart rate, prevalence of comorbidities (hypertension, diabetes mellitus, and cardiovascular disease), neutrophil count, lymphocyte count, platelet (PLT) count, procalcitonin (PCT), interleukin-6 (IL-6) levels, and Sequential Organ Failure Assessment (SOFA) scores, were extracted from electronic health records upon admission. Complete blood counts (neutrophils, lymphocytes, and PLT) were determined using the Maccura F81 fully automated hematology analyzer (Maccura Biotechnology, Sichuan, China). Serum PCT and IL-6 levels were quantified via electrochemiluminescence immunoassay using the Cobas 800 modular analytics platform (Roche Diagnostics, Rotkreuz, Switzerland). For samples that exceeded the assay’s upper detection limit for PCT (>100 ng/mL), a 101 ng/mL value was recorded.

### Statistical analysis

Continuous variables conforming to a Gaussian distribution are expressed as mean ± standard deviation and evaluated using independent samples t-tests. Non-Gaussian continuous variables are summarized as median with interquartile range and compared using the Mann–Whitney U test. Categorical data are presented as frequency counts (percentages) and compared using the χ² test. Inter-variable correlations between continuous parameters and LPA levels were assessed using Spearman’s ρ coefficient. Covariates demonstrating statistical significance (P < 0.05) in univariate logistic regression were incorporated into multivariate logistic regression using a forced-entry technique to determine the independent relationship of LPA with sepsis. The diagnostic and prognostic performance of LPA and other biomarkers (procalcitonin, PCT; neutrophil count) for sepsis identification and in-hospital mortality prediction was evaluated using receiver operating characteristic (ROC) curve analysis. The area under the ROC curve (AUC) along with its 95% confidence interval (CI) was calculated. The optimal cut-off value was determined by maximizing the Youden’s index. Comparisons of the AUCs between different biomarkers were performed using the DeLong test. Statistical significance was defined as a two-sided α-level of 0.05. All analyses were conducted using SPSS (version 26.0.0.0; IBM Corp., Armonk, NY).

## Results

### Study population characteristics

The study cohort comprised 71 participants, including 42 patients clinically diagnosed with sepsis (sepsis group) and 29 patients with non-septic infections (control group). In the sepsis group, 15 experienced in-hospital mortality. **Table 1** summarizes baseline characteristics of all participants. Compared with the control group, the sepsis group demonstrated significantly elevated values for body temperature, respiratory rate, neutrophil count, PCT, IL-6, and SOFA scores. Additionally, the sepsis group had significantly higher rates of positive blood culture and assisted ventilation compared to the control group. In contrast, they exhibited reduced lymphocyte and platelet counts. In the control group, non-survivors exhibited significantly higher heart rates and SOFA scores than survivors. Interestingly, plasma LPA levels were significantly lower in the sepsis group than in the control group, with non-survivors demonstrating further reductions than survivors with sepsis.

**Table 1:** Basic characteristics of study subjects by groups.

Variables	Control (n = 29)	Sepsis (n = 42)	^a^P	Sepsis		
				Survivors (n = 27)	Non-survivors (n = 15)	^b^P
Age (years)	78.0 (69.5, 84.0)	79.0 (72.8, 85.0)	0.788	78.0 (71.0, 83.0)	80.0 (77.0, 85.0)	0.207
Male, n (%)	16 (55.2%)	22 (59.5%)	0.715	16 (59.3%)	9 (60.0%)	0.963
BMI (kg/m^2^)	22.4 (19.9, 25.1)	21.4 (17.1, 26.2)	0.437	22.5 (19.3, 27.8)	19.5 (15.4, 23.2)	**0.049**
Temperature (°C)	36.8 ± 0.8	37.4 ± 1.0	**0.006**	37.4 ± 1.0	37.5 ± 1.1	0.741
Respiratory (rate/minute)	20.8 ± 0.7	23.2 ± 3.7	**< 0.001**	23.8 ± 4.1	22.0 ± 2.6	0.132
Heart rate (bpm)	89.0 (78.5, 110.5)	99.0 (84.8, 115.5)	0.128	90.0 (81.0, 102.0)	110.0 (104.0, 122.0)	**0.003**
Hypertension, n (%)	13 (44.8%)	26 (61.9%)	0.155	15 (55.6%)	11 (73.3%)	0.256
Diabetes, n (%)	23 (79.3%)	35 (83.3%)	0.667	24 (88.9%)	11 (73.3%)	0.195
Heart disease, n (%)	21 (72.4%)	32 (76.2%)	0.719	21 (77.8%)	11 (73.3%)	0.746
Positive blood culture, n (%)	7 (24.1%)	28 (66.7%)	**< 0.001**	20 (74.1%)	8 (53.3%)	0.172
Assisted ventilation, n (%)	9 (31.0%)	39 (92.9%)	**< 0.001**	24 (88.9%)	15 (100%)	0.180
Neutrophil (×10^9^ cells/L)	5.16 (3.73, 8.71)	8.37 (5.08, 13.79)	**0.031**	6.87 (4.25, 13.46)	10.92 (5.18, 20.23)	0.372
Lymphocyte (×10^9^ cells/L)	1.08 (0.79, 1.30)	0.83 (0.57, 1.17)	**0.034**	0.92 (0.61, 1.20)	0.73 (0.51, 1.12)	0.118
PLT (×10^9^ cells/L)	207.0 (171.0, 238.0)	143.0 (95.0, 243.5)	**0.018**	161.0 (116.0, 216.0)	120.0 (90.0, 300.0)	0.823
PCT (ng/ml)	0.23 (0.07, 1.66)	2.02 (0.59, 6.26)	**< 0.001**	1.97 (0.60, 21.04)	2.07 (0.55, 4.77)	0.813
IL-6 (pg/ml)	67.5 (17.2, 195.7)	141.4 (63.5, 449.7)	**0.021**	97.9 (23.1, 366.6)	196.6 (99.6, 901.8)	0.059
SOFA	1.0 (0, 2.0)	5.0 (3.0, 9.0)	**< 0.001**	4.0 (3.0, 7.0)	7.0 (5.0, 11.0)	**0.019**
LPA (ng/ml)	384.9 (365.9, 395.5)	329.4 (278.1, 351.7)	**< 0.001**	337.1 (313.8, 353.4)	289.1 (261.8, 329.6)	**0.004**

a P, value among the control and sepsis groups.

b P, value among the survivor, and non-survivor sepsis groups.

BMI, body mass index; PLT, platelet; PCT, procalcitonin; IL-6, interleukin 6; SOFA, sequential organ failure assessment; LPA, lysophosphatidyl acid.

Boldface highlights results with statistically significant differences.

### Correlation between LPA and clinical indicators

Spearman correlation analysis evaluated the relationship between plasma LPA levels and clinical/laboratory parameters (**Table 2**). LPA levels were inversely correlated with body temperature, respiratory rate, and heart rate (all P<0.01). Specifically, LPA levels were negatively correlated with major infection-related and inflammatory biomarkers, such as neutrophil count, PCT, and IL-6 (all P < 0.001). Conversely, LPA levels were positively correlated with lymphocyte and platelet counts (P < 0.05). No significant correlations were observed between LPA levels and age, BMI, or PLT count (all P > 0.05). Notably, a robust inverse correlation was identified between LPA levels and sepsis severity, as measured by SOFA scores (P = -0.706, P<0.001).

**Table 2:** Correlation between LPA and clinical indexes.

Variables	r	P
Age (year)	-0.064	0.598
BMI (kg/m^2^)	0.058	0.631
Temperature (°C)	-0.359	**0.002**
Respiratory (rate/minute)	-0.307	**0.009**
Heart rate (rate/minute)	-0.368	**0.002**
Neutrophil (×10^9^ cells/L)	-0.411	**< 0.001**
Lymphocyte	0.245	**0.040**
PLT (×10^9^ cells/L)	0.209	0.081
PCT (ng/ml)	-0.470	**< 0.001**
IL-6 (pg/ml)	-0.365	**0.002**
SOFA	-0.706	**< 0.001**

LPA, lysophosphatidyl acid; BMI, body mass index; PLT, platelet; PCT, procalcitonin; IL-6, interleukin 6; SOFA, sequential organ failure assessment.

Boldface highlights results with statistically significant differences.

### Relationship of LPA levels with the risk of sepsis and In-hospital mortality

To investigate the predictive performance of LPA, univariate and multivariate logistic regression analyses were conducted. Univariate analysis suggested that body temperature, respiratory rate, neutrophil count, PCT, and IL-6 emerged as significant risk factors for sepsis. After multivariate adjustment for these confounders, plasma LPA levels were identified as an independent predictor of sepsis (Odds ratio (OR) = 0.931, 95% confidence interval (CI) 0.893–0.971, P = 0.001). Furthermore, LPA remained independently associated with in-hospital mortality after adjusting for the mentioned covariates (OR = 0.950, 95% CI 0.921–0.980, P = 0.001) (**Table 3**).

**Table 3:** Relative risk of LPA for sepsis and mortality.

Univariate			Multivariate^#^	
Variables	OR (95% CI)	P	OR (95% CI)	P
Sepsis	0.934 (0.903 - 0.965)	**< 0.001**	0.931 (0.893 - 0.971)	**0.001**
Mortality	0.970 (0.955 - 0.986)	**< 0.001**	0.950 (0.921-0.980)	**0.001**

^#^Adjusted for body temperature, respiratory rate, neutrophil count, PCT, and IL-6.

LPA, lysophosphatidyl acid; PCT, procalcitonin; IL-6, interleukin 6.

Boldface highlights results with statistically significant differences.

### Clinical value of LPA in sepsis diagnosis and In-hospital mortality risk prediction

**Figure 1:**
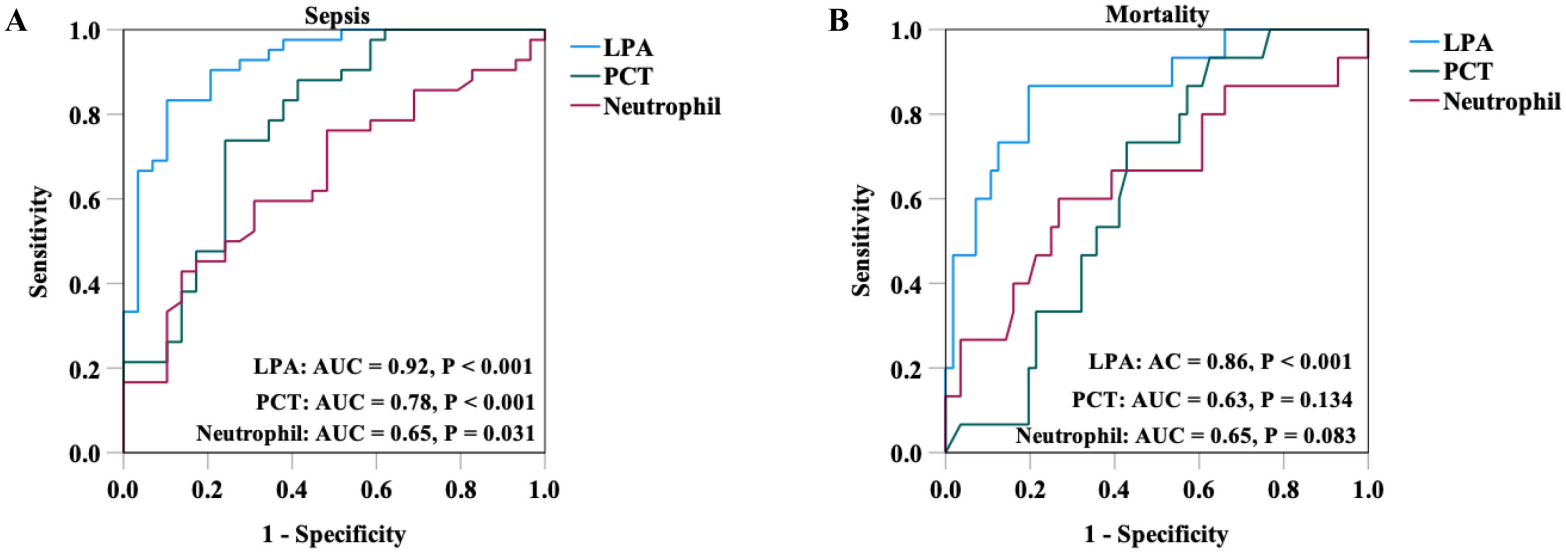
ROC curve of LPA, PCT and neutrophil in sepsis diagnosis **(A)** and in-hospital mortality prediction **(B)**. LPA, lysophosphatidyl acid; PCT, procalcitonin.

ROC curve analysis was conducted to evaluate the diagnostic and prognostic performance of LPA in sepsis and in-hospital mortality prediction. As shown in **Figure 1**, for sepsis diagnosis+, LPA demonstrated excellent discriminatory power with AUC of 0.92 (95% CI = 0.86–0.99, P < 0.001). At an optimal cut-off value of 355.57 ng/mL, it achieved 89.7% sensitivity and 83.3% specificity. Notably, when compared with other biomarkers, LPA exhibited superior diagnostic performance, as the AUC for PCT was 0.78 (95% CI: 0.66 - 0.89, P < 0.001) and for neutrophil count was 0.65 (95% CI: 0.52-0.78, P = 0.031). Furthermore, LPA showed strong prognostic value for predicting in-hospital mortality among septic patients, with an AUC of 0.86 (95% CI = 0.76–0.97, P < 0.001). The optimal threshold for mortality prediction was 333.40 ng/mL, corresponding to 80.4% sensitivity and 86.7% specificity. The prognostic performance of LPA was also superior to that of PCT (AUC = 0.63, 95% CI: 0.49 - 0.76, P = 0.134) and neutrophil count (AUC = 0.65, 95% CI: 0.47 - 0.82, P = 0.083).

## Discussion

The complex etiology, substantial morbidity and mortality, and persistent diagnostic challenges of sepsis continue to render it a formidable clinical problem. Sepsis is characterized by a dysregulated host response to infection, leading to systemic inflammation, multiorgan dysfunction, and potentially death, posing a significant burden on healthcare systems worldwide (16, 17). The differentiation of sepsis from other common illnesses with similar presentations, such as severe pneumonia, remains difficult (18). Current diagnostic standards, including blood culture, are hampered by prolonged turnaround times and variable sensitivity. While biomarkers like procalcitonin (PCT) and C-reactive protein (CRP) are routinely employed, recent meta-analyses highlight their limitations in diagnostic performance. For PCT, summary AUC values range from 0.74 to 0.88 across different patient populations, with sensitivity and specificity varying substantially (19–22). Similarly, CRP demonstrates variable performance with AUC values of 0.67 in general sepsis populations and up to 0.945 in pediatric cases, yet its specificity can be as low as 55% in some analyses (19, 23). These limitations can delay appropriate treatment, worsen patient outcomes, and contribute to antibiotic resistance (24, 25). Consequently, the identification of rapid, sensitive, and specific biomarkers is crucial for early diagnosis, risk stratification, and improved management of sepsis.

LPA exerts its biological effects primarily through specific G-protein-coupled receptors (LPA1-LPA6), influencing processes critical to inflammation and diseases such as asthma and acute lung injury (26). It is important to note that LPA’s effects can be context-dependent, exhibiting both pro- and anti-inflammatory properties. Nevertheless, a body of preclinical evidence, including work from our group, underscores its protective role in sepsis. In murine models, administering LPA or specific receptor agonists can suppress LPS-induced inflammation, inhibit harmful processes like neutrophil extracellular trap (NET) formation, and ultimately improve survival (13, 14, 27). However, the clinical relevance of these findings, particularly the dynamics of plasma LPA levels in septic patients and their correlation with disease severity and outcomes, has remained largely unexplored.

Our study provides this critical clinical validation by demonstrating that plasma LPA is significantly reduced in sepsis patients and is lowest in non-survivors. This finding aligns with the well-established consensus that levels of LPC, the major biosynthetic precursor for LPA, are consistently lower in septic patients and have prognostic value for mortality (28–30). The significant negative correlations we observed between LPA levels and established markers of infection (PCT) and inflammation (neutrophil count, IL-6) further suggest that LPA may function as a negative regulator of the inflammatory response in human sepsis, consistent with the anti-inflammatory effects reported in preclinical models (14, 15, 31). Furthermore, we established that low LPA levels are independently associated with both the diagnosis of sepsis and in-hospital mortality, with ROC curve analysis affirming its strong diagnostic and prognostic utility. The primary contribution of our work lies not in the discovery of LPA’s involvement in sepsis, but in providing comprehensive clinical evidence supporting its translation into a clinically useful biomarker.

Beyond establishing the diagnostic and prognostic value of LPA, its translation into clinical practice requires consideration of practical implementation. While mass spectrometry offers precise LPA speciation (32), its complexity limits routine use. The ELISA method used in our study provides a more viable alternative, with established standardization, simpler workflow, lower cost, and a turnaround time (2–3 hours) compatible with sepsis management. Integration into clinical workflows could involve measuring LPA at admission alongside SOFA scores to enhance early risk stratification and guide treatment decisions. However, multi-center studies remain necessary to standardize assays, define optimal cut-off values, and validate LPA-guided protocols.

The observed reduction in plasma LPA presents a paradox when considering the complex regulation of its metabolism. The enzyme autotaxin (ATX) is the primary catalyst for LPA production from LPC. Interestingly, one clinical study reported elevated circulating ATX levels in sepsis patients (33), a finding that might ostensibly conflict with our observation of low LPA. This apparent contradiction suggests that the net plasma concentration of LPA is not determined by ATX levels alone but is likely a balance of its synthesis and consumption. We therefore hypothesize that in human sepsis, the elevated ATX might represent a compensatory response, while the observed net decrease in LPA could be primarily driven by its accelerated consumption. This consumption may occur through heightened signaling via LPA receptors on immune cells, mediating its anti-inflammatory effects, and/or through other clearance pathways. This model helps reconcile conflicting reports in the literature. For instance, a murine model of polymicrobial sepsis reported decreased levels of both LPC and ATX alongside an increase in LPA (34), highlighting that the dynamics of the LPC-ATX-LPA axis are profoundly influenced by the specific experimental or clinical context. Our clinical data firmly establish that low plasma LPA is a feature strongly associated with adverse outcomes in human sepsis. The validation of this consumption-centric hypothesis and the precise mechanisms involved warrant further investigation.

The novelty of our study lies in the identification of plasma LPA as a promising dual-purpose biomarker for sepsis diagnosis and in-hospital mortality risk prediction. However, several limitations warrant careful consideration. First, the single-center design and relatively modest sample size limited our statistical power for detailed subgroup analyses and multivariable adjustments for potential confounders, which may affect the generalizability of our findings. Future larger-scale studies are needed to validate our results. Second, the measurement of LPA at a single early time point prevents the assessment of its kinetic profile during disease progression and in response to treatments; longitudinal studies with serial measurements are warranted. Third, this study focused specifically on total LPA based on existing preclinical evidence of its anti-inflammatory role, and we did not investigate a broader panel of lipidomic mediators or LPA subspecies. Comprehensive lipidomic profiling in future studies could provide a more complete understanding of lipid metabolism in sepsis. Finally, while our study provides clinical evidence of LPA’s biomarker potential, the precise mechanisms underlying LPA reduction in sepsis remain to be fully elucidated.

## Conclusion

This study provides initial clinical evidence supporting plasma LPA as a potential biomarker in sepsis. We demonstrate that reduced LPA levels are characteristic of sepsis patients and correlate inversely with disease severity and key inflammatory markers. Most importantly, LPA exhibits promising predictive value for both sepsis diagnosis and in-hospital mortality, suggesting its potential as a complementary tool for early risk stratification. However, future large-scale, prospective studies are needed to confirm these findings and establish the clinical applicability of LPA in sepsis management. .

## Data Availability

The original contributions presented in the study are included in the article/**Supplementary Material**. Further inquiries can be directed to the corresponding authors.
