# Supplementary material for: Lysophosphatidic acid: a promising biomarker for diagnosing sepsis and predicting in-hospital mortality
Source: Front Immunol. 2026 Jan 6;16:1725394. doi: 10.3389/fimmu.2025.1725394 (PMC12816347; doi:10.3389/fimmu.2025.1725394)
Supplement: Supplementary file 1 [file Supplementaryfile1.docx]

**Methods for LPA Measurement**

1. **Detailed Protocol for LPA Measurement by ELISA**

The detailed, step-by-step procedure for quantifying total plasma LPA levels using the commercial ELISA kit (LPA Assay Kit II, cat# K-2800S, Echelon Biosciences) is described below. The procedure was performed exactly as per the manufacturer's instructions.

**1.1 Reagent and Plate Preparation:**

All standards, detection reagents, and wash buffer were prepared according to the kit specifications prior to the assay. The pre-coated microplate was brought to room temperature.

**1.2 Assay Procedure:**

1. Addition of Samples and Standards: 50 µL of each plasma sample (diluted 1:2 as recommended), standard, and blank control were added to the designated wells in duplicate.
2. Addition of Detection Antibody: 50 µL of Detection Reagent A (biotin-conjugated anti-LPA antibody) was immediately added to each well.
3. First Incubation: The plate was sealed and incubated for 1 hour at 37°C.
4. Washing: Following incubation, the sealing film was removed, and the liquid was discarded. Each well was washed five times with 300 µL of the provided Wash Buffer. After the final wash, the plate was inverted and blotted firmly on clean absorbent paper to remove residual liquid.
5. Addition of Streptavidin-HRP: 100 µL of Detection Reagent B (Streptavidin-Horseradish Peroxidase conjugate) was added to each well.
6. Second Incubation: The plate was sealed and incubated for 30 minutes at 37°C.
7. Washing: The washing procedure (Step 4) was repeated exactly for a total of five washes.
8. Substrate Reaction: 90 µL of Substrate Solution (TMB) was added to each well. The plate was incubated for 15 minutes at 37°C in the dark.
9. Reaction Termination: 50 µL of Stop Solution was added to each well. The blue color changed to yellow immediately.
10. Absorbance Measurement: The optical density (OD) of each well was measured at a wavelength of 450 nm using a microplate reader within 10 minutes after adding the stop solution.

**1.3 Data Calculation:**

The average OD value for the blank control was subtracted from all standard and sample readings. A standard curve was generated by plotting the average OD value for each LPA standard against its known concentration. The LPA concentration in each plasma sample was then interpolated from this four-parameter logistic (4-PL) curve.

**2. Antibody Specificity and Cross-reactivity**

The monoclonal antibody (504B3/B3) used in the LPA Assay Kit II is highly specific for LPA. As per the manufacturer's validation data, the antibody exhibits minimal cross-reactivity with structurally similar lipids. The key cross-reactivity data is summarized in Table S1 below.

Table S1. Cross-reactivity Profile of the Anti-LPA Antibody (504B3/B3)


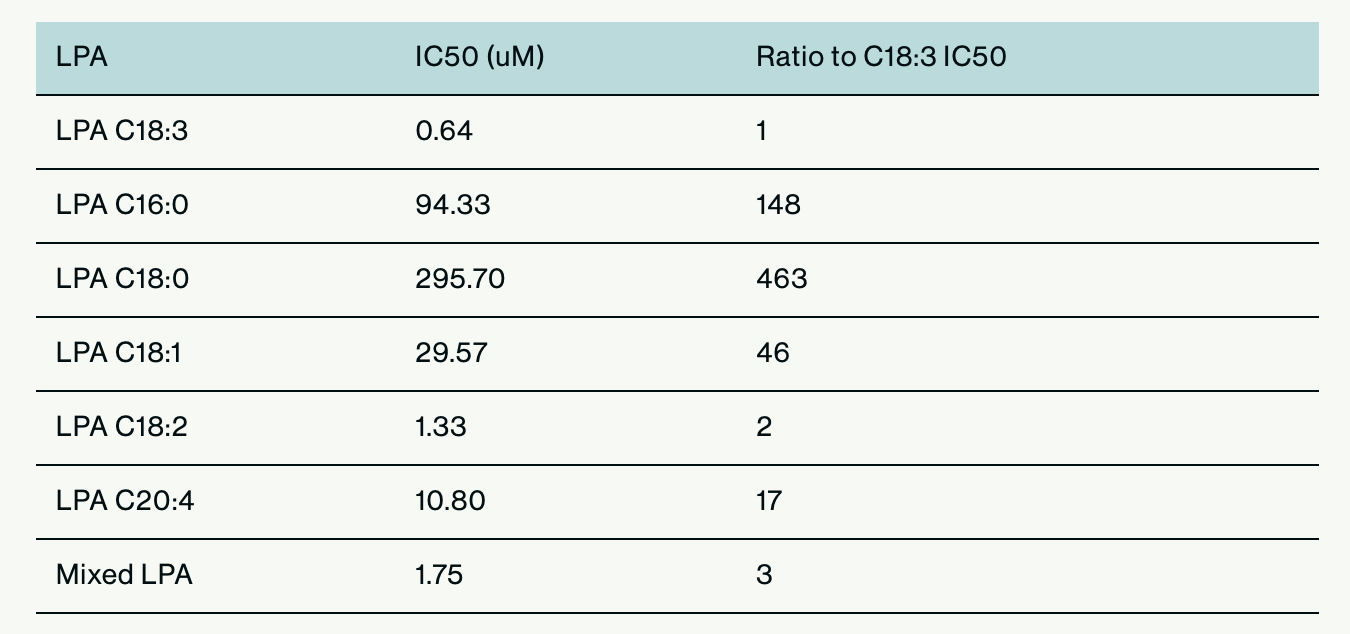


Furthermore, the antibody recognizes a broad spectrum of LPA species with varying affinity. Its relative sensitivity toward different LPA species is as follows, from highest to lowest: 18:3 ≈ 18:2 > 18:1 > 18:0 > 20:4 > 16:0. This profile ensures comprehensive detection of the major LPA species present in human plasma. The full validation data can be accessed on the manufacturer's website: <https://www.echelon-inc.com/how-specific-is-the-lpa-assay-kit-ii/>
